# Paediatric kidney stone surgery: state-of-the-art review

**DOI:** 10.1177/17562872231159541

**Published:** 2023-03-18

**Authors:** Patrick Juliebø-Jones, Etienne Xavier Keller, Lazaros Tzelves, Christian Beisland, Bhaskar K Somani, Peder Gjengstø, Mathias Sørstrand Æsøy, Øyvind Ulvik

**Affiliations:** Department of Urology, Haukeland University Hospital, Jonas Lies vei 65, 5021 Bergen, Norway; Department of Urology, University Hospital Zurich, University of Zurich, Zurich, Switzerland EAU YAU Urolithiasis Group, Arnhem, The Netherlands; Second Department of Urology, National and Kapodistrian University of Athens, Sismanogleio General Hospital, Athens, Greece EAU YAU Urolithiasis Group, Arnhem, The Netherlands; Department of Urology, Haukeland University Hospital, Bergen, NorwayDepartment of Clinical Medicine, University of Bergen, Bergen, Norway; Department of Urology, University Hospital Southampton, Southampton, UK; Department of Urology, Haukeland University Hospital, Bergen, Norway; Department of Urology, Haukeland University Hospital, Bergen, Norway; Haukeland University Hospital, Bergen, NorwayDepartment of Clinical Medicine, University of Bergen, Bergen, Norway

**Keywords:** endourology, paediatric, technology, ureteroscopy, urolithiasis

## Abstract

While urolithiasis in children is rare, the global incidence is rising, and the volume of minimally invasive surgeries being performed reflects this. There have been many developments in the technology, which have supported the advancement of these interventions. However, innovation of this kind has also resulted in wide-ranging practice patterns and debate regarding how they should be best implemented. This is in addition to the extra challenges faced when treating stone disease in children where the patient population often has a higher number of comorbidities and for example, the need to avoid risk such as ionising exposure is higher. The overall result is a number of challenges and controversies surrounding many facets of paediatric stone surgery such as imaging choice, follow-up and different treatment options, for example, medical expulsive therapy, shockwave lithotripsy, ureteroscopy, and percutaneous nephrolithotomy. This article provides an overview of the current status of paediatric stone surgery and discussion on the key topics of debate.

## Introduction

Kidney stone disease (KSD) is not common among children, but the global incidence is increasing and is estimated to lie between 1% and 15% depending on geographic location and epidemiological source.^1,2^ Regarding sex distribution, it is commonest in men in the first decade of life compared with women in the second. Interestingly, findings from longitudinal studies reveal that across both adult and paediatric population groups, the greatest rise has been among adolescent women.^3,4^ Up to 30% of presenting paediatric patients will require surgical intervention.^5^ Given the risk of symptomatic recurrence, reported as high as 50% at 3 years, the need to deliver treatments with maximal efficacy and minimal morbidity is of paramount importance.^6^ The challenge is heightened further in this special population given by the proportion of these patients with concomitant and often complex medical comorbidities.^7^ As highlighted by Ellison and Yonekawa,^8^ a selected intervention should allow for quick return to normal functions of daily living, no risk of long-term injury to the renal unit(s), high success rate in a single procedure, low to minimal radiation exposure and avoidance of anaesthetic exposure if at all possible. However, it is the case that the evidence basis driving clinician decision-making in this nonindexed patient group is largely made up of studies, which do not hold the highest levels of evidence, that is, retrospective and single-centre studies.^9^ Indeed, only three randomised trials are currently cited as references in the European guidelines.^10^ Operative strategies are often the result of adaptations from techniques developed in the adult setting, such as surgical experience with ureteral access sheaths (UASs) for example. The net result is that there currently exist numerous challenges and controversies in the setting of paediatric KSD management. This review aims to provide an overview and evaluation of these issues and provide the reader with a better understanding to help guide their clinical practice accordingly.

## Materials and methods

A comprehensive, nonsystematic review was performed to identify relevant literature addressing the treatment and management of paediatric stone disease. All study types were considered. Bibliographic databases searched included MEDLINE, Google Scholar and the Cochrane library. Search terms included ‘paediatric’, ‘urolithiasis’, ‘stones’, ‘shockwave lithotripsy’ and ‘percutaneous nephrolithotomy’ among others. The following key topics were identified: imaging, medical expulsive therapy (MET), shockwave lithotripsy (SWL), ureteroscopy (URS), percutaneous nephrolithotomy (PCNL) and delivery of care.

## Imaging

Ultrasound (US) is currently recommended as first-line imaging modality of choice in children, with a sensitivity and specificity for detection of renal stones of 61–93% and 95–100%, respectively.^11^ It can also be combined with plain x-ray. Noncontrast computed tomography (NCCT) is commonly used for surgical planning in more complex cases, which may have more difficult anatomy and large stone burdens. As well as higher sensitivity (97–100%) and specificity (96–100%), NCCT holds a further benefit of assessing stone density, which also aids operating planning.^12,13^ These advantages must be balanced with radiation-associated risks, particularly the risk of fatal cancer from paediatric NCCT.^14^ This shortcoming is of particular relevance to patients with high risk of stone recurrence, such as patients with cystinuria. Data from the United States reveal that a surprisingly high proportion (>60%) of children in fact undergo NCCT as the initial imaging modality.^15^ Another US study of >2500 paediatric patients undergoing URS revealed that NCCT is the commonest (71%) imaging modality at follow-up.^16^ Ultra-low-dose protocols (0.5 mSv) can be used to mitigate radiation exposure. Magnetic resonance (MR) imaging using gadolinium to provide an excretory phase is used rarely (<3%), and while it carries high sensitivity (up to 100%), it is limited by high costs, motion artefacts and anaesthetic requirement.^13,17^ Nuclear medicine renal scans can also be performed in patients with upper urinary tract stone and hydronephrosis to distinguish concomitant pelvic-ureteric junction obstruction (PUJO).

### Use of nomograms

Predictive nomograms, which incorporate factors based on imaging, for example, location and stone size, have gained increasing attention to ameliorate operative planning and patient counselling.^18,19^ However, while certain nomograms have been developed for use specifically in the paediatric setting, others have been developed in adults using NCCT but then applied to paediatric patients where US or plain x-ray is used.^20^ NCCT also allows for stone volume measurement, which is arguably a more accurate means of reporting stone size as well as, for example, measuring laser efficiency.^21^

### Reducing radiation exposure

Regarding intraoperative efforts to reduce ionising radiation exposure, surgeons can consider totally ultrasonographic guided PCNL in children.^22^ Fluoroless URS has been described in the adult setting, but these are usually noncomplex cases and experienced endourological centres.^23^ Gonad protection can be used during procedures, but a recent survey highlighted that clinicians use it routinely in less than 50% of cases.^24^

## Treatment options

### MET

Conservative treatment for small asymptomatic stones <5 mm is indicated where there is a possibility of spontaneous passage. In Europe, MET is an off-licence option for use in children. Several meta-analyses have shown that overall, it seems to have a beneficial effect on stone expulsion in children, especially in distal ureteric stones.^25,26^ However, current guidelines highlight the limitations, bias and imprecision in the included trials, and they make no formal recommendations on MET in this patient category. Despite this uncertainty, its use has risen in the United States.^5^ Tamsulosin has also been studied as a preoperative strategy to lower the access failure rate at the time of URS with or without UAS placement. Multiple single-centre and retrospective studies have shown it to have beneficial effect, but further prospective and randomised studies are warranted to verify these initial findings.^27,28^

### SWL

While the global trends have seen SWL declining in its use, guidelines do still support this intervention as being as the first-line treatment of choice for most paediatric ureteral stones and is also an option for renal stones up to 2 cm in size if the anatomy is determined to be amenable.^29^

#### Anaesthesia

A disadvantage of SWL in the paediatric population can be the requirement for general anaesthesia (GA) (Table 1). Practice patterns for anaesthetic approach vary globally but most will generally require this anaesthetic type.^30^ First-generation machines deliver more energy and higher fragmentation rate but at a cost of more patient discomfort and hence requirement for GA.^10,31^ Older patients and with use of later generation machines, which deliver less energy at a cost of smaller focal zone, are better candidates for sedative anaesthesia. However, it is worth noting that at present, there are very few studies reporting experiences with such an anaesthetic approach.^32^

**Table 1.** Success rates, advantages and disadvantages of different minimally invasive procedures.

Modality	Overall stone-free rate	Advantages	Disadvantages
SWL	70–90%	• Least invasive intervention • IV sedation/PCA (or no sedation possible in older children) • Short learning curve	• Most paediatric cases still require general anaesthesia • Reduced success in stones >10 mm in diameter • More likely to require retreatment • Not suitable for impacted stones and unfavourable pelvicalyceal anatomy • Lower success in cystine stones and calcium monohydrate stones • Retreatment, auxiliary procedures often needed
URS	76–100%	• Shorter hospital stay than PCNL • Improved complication profile compared with PCNL Better stone-free rate compared with SWL	• Failure to access the renal unit during primary URS can occur, requiring prestenting • Post-URS stent often done, often needing general anaesthesia for removal
PCNL	86.9–95%	• Best suited for large stones • Most likely to achieve success in a single session • Miniaturised instruments allow reduced complication rates • Can use ultrasound to reduce radiation exposure • Favourable option where URS and SWL have failed	• Higher complication rate, compared with SWL or URS • Longest learning curve, compared with SWL or URS • Longer hospital stay

IV, intravenous; PCA, patient-controlled analgesia; PCNL, percutaneous nephrolithotomy; SWL, shockwave lithotripsy; URS, ureteroscopy.

#### Stenting during SWL

Practice also varies for placing ureteral stent during SWL treatment. Previous studies have shown that while this does not improve stone-free rate (SFR), it can lower complications and hospital stay.^31,33^ It is therefore a worthwhile consideration in patients with large stone burdens at high risk of steinstrasse.^34^ Ureteral stent placement in children necessitates removal under GA but novel methods such as stent on string or use of magnetic retrieval device have been reported in studies to be tolerated under local anaesthesia (LA) and with a high success rate.^35^

#### URS

Compared with SWL, there have been many more developments related to the techniques and technology of URS. These include next-generation and miniaturised digital scopes, new laser platforms such as thulium fibre laser (TFL) as well as innovations in pulse modulation such as Moses technology.^36^-^38^ These developments have contributed to the marked rise in the number of paediatric URS series which have been published, as well as the steady improvement in SFRs, which have been reported over recent years.^7,12,39,40^ These advancements have also supported the use of URS for difficult scenarios such as lower pole stones and cystinuria.^41,42^

#### Energy sources for lithotripsy

Ho:YAG lasers represent mainstay of energy source used in studies to date, although pneumatic/ballistic sources are still widely used in the clinical setting. Indeed, the Clinical Research Office of the Endourological Society (CROES) reported use of the latter in >85% of URS treatments for ureteral stones.^43^ Regarding machine power, Kaygısız *et al.*^44^ recently compared outcomes between the use of 15- and 30-W Ho:YAG laser generators and found the latter to achieve shorter operative times (40 min *versus* 52.5 min, *p* < 0.05). To date, there have only been two studies addressing TFL in the paediatric setting.^45,46^ First, a retrospective comparative study by Jaeger *et al*., which found SFR to be lower in the TFL group compared with Ho:YAG (70% *versus* 59%, *p* < 0.05). Second, a recent global study on paediatric URS recorded that TFL was associated with shorter operative times.^46^ These benefits are welcomed in the paediatric setting, given the priority of reducing operative time, which has been previously identified as an independent predictor of complications.^47^

#### Miniaturisation of instruments

Another development, which appears to translate well to the paediatric setting, is the miniaturisation of ureteroscopes. These have partly come in the form of single-use flexible ureteroscopes such as Uscope 7.5 Fr (Pusen Ltd., Zhuhai, China).^36^ Single-use scopes may offer advantages in cases with difficult anatomy and therefore high risk of scope damage and cases of multiresistant urinary infection.^48^ However, the cost efficacy of such devices remains in question as does their true environmental impact.^49^ Another important consideration is that in some of these single-use models, the tip and shaft of the ureteroscope are of the same diameter and the tip is not tapered. This is related to limitations regarding the smallest size possible that a digital scope can be constructed given the processor (i.e. the chip) is located at the tip. In contrast, nondigital reusable scopes are available with smaller tips, for example, 4.9 Fr. In a recent study by Kahraman *et al.*,^12^ use of scopes with this sized tip allowed for higher SFR. Similarly, use of a ‘mini’ semirigid ureteroscope (4.5 Fr) has also gained attention with studies revealing higher treatment success rates for ureteral stones with this sized scope.^50^ These miniaturisations can also potentially serve to reduce need for prestenting.^51^

#### Role of UAS

**Figure 1.**
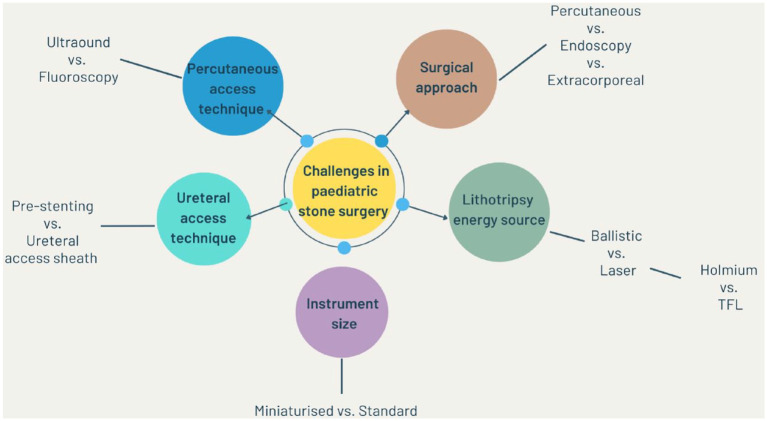
Overview of challenges in paediatric stone surgery.

An increasing number of series examining the role of UAS have been reported, and a recent multicentre study recorded their use is now routine in more than half of the cases performed.^46,52,53^ This accessory serves to facilitate access as to the upper urinary tract and improve irrigation with the additional aims of reducing intrarenal pressure and temperature. Their use divides opinion, and arguments against their use include risk of ureteral stricture, damage to ureteroscope and postoperative pain^54^ (Figure 1). In a series of 96 paediatric patients, Wang *et al.*^52^ reported an increased rate of complications in the intraoperative setting but not at long-term follow up. However, use of UAS in that particular study did not lead to any significant difference in the SFR. One measure to the reduced risk of injury associated with UAS is to use a smaller size, for example, 10/12 Fr.^53,55^ Even though the larger UASs have been repeatedly associated with lower intrarenal pressure and temperature,^56,57^ one should rather consider the ratio or the cross-sectional area between the ureteroscope and the UAS, which would take into consideration the space available for irrigation fluid outflow.^58,59^ As a matter of fact, the use of a 7.5-F scope with a 10/12-F UAS would theoretically achieve a similar pressure and temperature reduction compared with a 10-F scope with a 12/14-F UAS because the available space between the scope and the inner wall of the UAS would be almost the same in both scenarios (3.8 mm^2^) and it is this space that allows for irrigational outflow (Table 2).

**Table 2.** Instrument dimensions.

Instrument	URS size (cross-sectional area)	UAS (cross-sectional area)	UAS/URS ratio	Available surface for irrigation outflow, mm^2^
Miniaturised instruments	7.5 F (4.909 mm^2^)	10/12 F (8.727 mm^2^)	1.8	3.818
Older generation instruments	10 F (8.727 mm^2^)	12/14 F (12.566 mm^2^)	1.4	3.839

UAS, ureteral access sheath; URS, ureteroscopy.

### PCNL

PCNL is the gold standard treatment for stones larger than 2 cm in the paediatric setting. However, as a therapeutic option, it historically holds disadvantages regarding its complication profile.

#### Miniaturisation of instruments

One area of attention has been the issue of using adult PCNL equipment (>22 Fr, also termed ‘maxi-PCNL’) in children, especially in lower age groups.^60^ One of the inaugural studies on this topic came from Jackman *et al.*,^61^ where the authors reported their experiences of using a 11-Fr vascular access sheath in a series of 11 children. In this study, they highlighted the analogy that given the smaller size difference in kidney, use of a 24-Fr sheath in a child is the equivalent of using a 72-Fr sheath in an adult.^61^ Since then, further advancements and refinements in PCNL miniaturisation have led to improvements regarding risks such as bleeding and shorter operative times.^62,63^ Choice of instrument can be tailored according to patient age and stone burden.^64^ Exact definitions for sizes do vary but generally, the following size categories apply: mini (16–22 Fr), super-mini (14–16 Fr), ultra-mini (10–13 Fr) and micro (4.85 Fr).

Rehman *et al.* recently reported 91% SFR in a report of 135 paediatric procedures (mean stone burden: 2.1 cm) undergoing mini-PCNL. Indeed, this approach is now being increasingly used by some centres as their standard PCNL approach in children.^65^ Miniaturisation also increases feasibility for tubeless exit strategies, which results in reduction in pain and hospital stay.^30,66^ Limitations of miniaturisation in PCNL have been issues of reduced visibility, difficulty extracting fragments and higher intrarenal pressures.^67^ The clinical introduction of vacuum-assisted evacuation sheaths may help overcome these issues as well as potentially reduce operative time.^68,69^ Referred to as semiclosed circuit vacuum-assisted mini-PCNL (vmPCNL) system, there currently exist a limited number of studies reporting use in the paediatric setting.^67,70^

Such are the advancements in paediatric PCNL that successful treatment of stones in solitary kidney and same-session bilateral PCNL have been reported.^71,72^ Regarding the latter, there currently only exist a handful of reported series and it is generally agreed that if performed, it should be in a highly experienced setting with strict patient selection criteria.^73,74^

#### Endoscopic combined intrarenal surgery

PCNL can be combined with URS in what is known as endoscopic combined intrarenal surgery (ECIRS), but there only exist several case reports of this approach in the paediatric setting.^75^ The authors anticipate this technique combined with miniaturised instruments will play a growing role in this age group in the future. There is also still a role for pyelolithotomy (e.g. robot assisted) in cases of complex stone disease refractory to the abovementioned methods and also combined with pyeloplasty when indicated.^76^

## Delivery of care

There exists wide variation in the provision of paediatric urology care regarding which specialty (paediatric nephrologist, paediatric surgeon or adult urologist) plans treatment. This can affect the volume of different treatments performed. For example, a recent survey of practice patterns by Önal *et al.*^77^ found that paediatric nephrologists would consistently opt for SWL as the initial treatment strategy across a range of clinical situations, whereas urologists would select URS or PCNL. Similarly, depending on the setting and location, variation exists regarding which speciality performs surgery (adult endourologist or paediatric urologist).

Some smaller nations, for example, Norway, rely on paediatric endourology cases being delivered by adult endourologists. Studies show that the transfer of skills for an experienced adult endourologist to perform URS is very achievable and has a short learning curve.^78^ However, Wang *et al.*^79^ reported that up to 60+ cases can be required for an adult endourologist to achieve competence at paediatric PCNL. Adopting a ‘twin surgeon’ model whereby an adult endourologist and paediatric surgeon operate together has been one approach to overcome this challenge.^80^

### Case volume and outcomes

A widely reported observation across many surgical fields has been that high-volume centres deliver superior outcomes. Interestingly, however, in the context of paediatric URS, a recent meta-analysis revealed that similar results were achieved between both medium-volume (>50 cases per year) and high-volume (>100 cases per year) centres.^81,82^ This arguably further supports the impression that gaining competency at paediatric URS is achievable for experienced adult endourologists.

### Equipment availability

Adherence to guidelines is also affected by equipment availability. A recent survey of European Association of Urology (EAU) members from 87 countries on paediatric stone management reported the following rates of treatment modality availability: SWL: 88%, flexible URS: 80%, standard PCNL: 92% and mini-PCNL: 66%.^24^

### Reporting standards

It is recommended to use a validated grading tool such as the modified Clavien tool when reporting complications.^83^ However, it is worth bearing in mind that even this tool was not created for a paediatric population and as highlighted in a study by Dwyer *et al.*,^84^ it holds less reliability in this patient group. Development of a purpose-designed paediatric complication grading tool is therefore warranted. Another factor to consider is that in certain nations, for example, the United Kingdom, conditions such as sepsis in a child automatically result in admission to intensive care (Clavien 4), whereas this is not always the case in other healthcare systems.

The Paediatric Ureteroscopy (P-URS) checklist is a newly developed tool to aid the reporting and reviewing of studies in the setting of paediatric URS.^85^ This offers an overview of key items and areas to be included in such studies with the aim of standardising the parameters reported and aid when comparing studies.

## Future developments

### Patient-reported outcomes

In adult stone surgery, the role of patient-reported outcome measures (PROMs) has become more recognised, and several validated and stone-specific tools are available.^86,87^ However, these remain lacking in the paediatric setting. It is anticipated that the Paediatric Kidney Stone Surgery (PKIDS) trial will be a valuable step towards addressing this gap.^88^

### Follow-up imaging

As well as reporting subjective outcome measures and complications, further attention is needed to improve standardisation of imaging follow-up. A recent study of >4000 children undergoing interventions for KSD revealed that only 63% had undergone any imaging at 3-month follow up.^16^

## Conclusion

There currently exist many challenges and consequently controversies in the setting of paediatric kidney stone surgery. These are present in all stages of the treatment pathway from assessment and planning to follow-up and of course, the surgery itself. Miniaturisation in both URS and PCNL has allowed for the safety and efficacy profile of these interventions to be improved in the paediatric setting. This has been complemented by technological advances in laser energy sources used for stone lithotripsy. Further research is needed to focus on development of PROMs in the paediatric setting and complication grading tools that are tailored to the paediatric setting.

## Declarations

**Ethics approval and consent to participate:** Not applicable.

**Consent for publication:** Not applicable.

**Availability of data and materials:** Not applicable.
